# Factors Associated with a Disposition-Based Indicator of Severe Injury Among Older Adults with Fall-Related Injuries: A Secondary Analysis of the 2023 Emergency Department-Based Injury In-Depth Surveillance

**DOI:** 10.3390/healthcare14172851

**Published:** 2026-09-04

**Authors:** Jeong-Suk Kim, Sook Kang

**Affiliations:** 1 Department of Nursing, Nambu University, Gwangju 62271, Republic of Korea; 2 Department of Nursing, Chosun Nursing College, Gwangju 61453, Republic of Korea

**Keywords:** aged, accidental falls, emergency service, hospital, injury severity, patient disposition, emergency department-based injury in-depth surveillance

## Abstract

**Highlights:**

**What are the main findings?**

- Older age, fall type, injury location, transportation mode, and level of consciousness were associated with a disposition-based indicator of severe injury among older adults with fall-related injuries.
- Emergency transportation and impaired consciousness showed relatively strong associations with the disposition-based indicator, although some associations varied across alternative outcome definitions.

**What are the implications of the main findings?**

- Emergency department disposition and physiological injury severity may reflect different aspects of fall-related injury in older adults.
- Injury characteristics and initial clinical status should be considered when interpreting the clinical disposition of older adults with fall-related injuries.

**Abstract:**

**Background/Objectives**: Falls are a major cause of injury-related morbidity and mortality among older adults. This study aimed to examine factors associated with a disposition-based indicator of severe injury among older adults with fall-related injuries using data from the 2023 Emergency Department-based Injury In-depth Surveillance. **Methods**: This secondary analysis used data from the 2023 Emergency Department-based Injury In-depth Surveillance conducted by the Korea Disease Control and Prevention Agency. A total of 24,632 adults aged ≥65 years with fall-related injuries were included. The primary outcome was a disposition-based indicator of severe injury, defined as hospital admission, transfer to a higher-level hospital, or death. Multivariable logistic regression was performed, and sensitivity analyses were performed using alternative disposition-based outcomes, Revised Trauma Score (RTS)-based physiological severity, and the original four AVPU categories. **Results**: Of 24,632 participants, 9248 (37.5%) met the criteria for the primary outcome. Older age, falls from a height, outdoor injuries, emergency transportation, and impaired consciousness were associated with higher odds of the primary outcome, whereas alcohol use at the time of injury, stair-related falls, injuries in road or traffic areas or commercial or public facilities, sports or leisure activities, other activities, and nighttime injuries were associated with lower odds. Emergency transportation and impaired consciousness were strongly associated with the outcome. Emergency transportation remained consistently associated across sensitivity analyses, whereas worsening AVPU levels showed progressively higher odds. **Conclusions**: Several patient, injury-related, and clinical characteristics were associated with the disposition-based indicator of severe injury. Variation across alternative outcome definitions highlights the importance of distinguishing between emergency department disposition and physiological injury severity.

## 1. Introduction

The aging population worldwide has made falls among older adults an increasingly important public health concern [1]. Falls are one of the most common causes of unintentional injury in older adults and a leading cause of injury-related morbidity and mortality, resulting in fractures, traumatic brain injuries, functional decline, and disability [2]. According to a recent Global Burden of Disease study, approximately 45.66 million falls occurred among adults aged ≥65 years worldwide in 2021, and this number is projected to continue increasing in the coming years [3]. In the United States, approximately 3 million emergency department (ED) visits and 1 million hospitalizations related to falls among older adults occur annually, indicating that falls are closely associated with increased healthcare utilization [4].

Older adults who experience falls may require substantial healthcare resources beyond initial ED treatment, including hospitalization, skilled nursing facility care, and increased mortality [5]. A community-based study reported that approximately 20% of older adults who received medical treatment for falls were subsequently hospitalized, with hip injuries, head injuries, and fractures closely associated with hospitalization [6]. Another study found that older adults with recurrent falls experienced more hospitalizations than those without a history of falls, and this pattern persisted during both short- and long-term follow-up [7]. A one-year follow-up study of older adults transported to the hospital by emergency medical services after a fall demonstrated substantial increases in mortality and healthcare use. The study also reported that factors identifiable during the initial assessment, including severe traumatic brain injury, pre-existing functional impairment, and comorbidity burden, were associated with subsequent mortality [5]. A recent study using U.S. hospital discharge data also reported that fall-related hospitalizations were associated with prolonged length of stay and increased healthcare costs, while patients with dementia were more likely to experience fall-related hospitalization and non-routine discharge, including transfer to skilled nursing facilities or home healthcare services [8]. These findings suggest that injury severity and clinical characteristics among older adults with fall-related injuries are closely associated with subsequent clinical outcomes and healthcare use.

Clinical outcomes following falls in older adults are influenced not only by the occurrence of the fall itself but also by patient characteristics, injury characteristics, and circumstances surrounding the injury. Previous studies have identified the location of falls as a factor associated with mortality [9]. Among hospitalized older adults with fall-related injuries, injury severity has been identified as a major predictor of adverse clinical outcomes, including intensive care unit admission, prolonged hospital stay, and mortality [10]. Injury patterns and severity may also differ according to the environment in which the injury occurs, such as indoor or outdoor settings [11]. Therefore, information available during the initial ED assessment has been recognized as an important component of risk stratification and treatment decision-making for older adults with fall-related injuries [12].

Previous studies have reported associations of health and injury characteristics with emergency department (ED) visits, hospitalization, and long-term outcomes among older adults with fall-related injuries; however, many have focused on specific populations, such as community-dwelling older adults, patients transported by emergency medical services, or patients admitted to individual healthcare institutions [5,6,10]. Korean studies using the Emergency Department-based Injury In-depth Surveillance data have also examined intracranial injuries following falls at home and the association between alcohol use and severe injury among older adults with ground-level falls [13,14], but these studies focused on specific fall circumstances or clinical outcomes. Therefore, evidence remains limited regarding the associations between ED disposition and a broader range of patient characteristics, injury circumstances, healthcare utilization, and initial clinical characteristics among older adults presenting to EDs with different types of fall-related injuries. The Emergency Department-based Injury In-depth Surveillance is a nationwide multicenter ED-based injury surveillance system that systematically collects information on these characteristics and clinical outcomes, providing a valuable data source for examining these associations [15].

Emergency department (ED) outcomes among older adults with fall-related injuries may be associated with multiple factors, including sociodemographic characteristics, fall mechanisms and environments, initial post-injury clinical status, and healthcare use characteristics. Accordingly, this study considered sociodemographic characteristics, fall mechanisms, environmental and situational characteristics of the injury, and initial clinical and healthcare use characteristics as relevant factors. Using data from the 2023 Emergency Department-based Injury In-depth Surveillance, this study aimed to examine the associations between these characteristics and a disposition-based indicator of severe injury among older adults with fall-related injuries. The findings may provide evidence for understanding the associations between patient and injury characteristics and ED outcomes in this population.

## 2. Materials and Methods

### 2.1. Study Design and Participants

This study was a secondary analysis of data from the 2023 Emergency Department-based Injury In-depth Surveillance conducted by the Korea Disease Control and Prevention Agency (KDCA). The surveillance is a hospital-based injury surveillance system conducted at 23 participating hospitals. The surveillance period for the data used in this study was from 1 January to 31 December 2023. The participating hospitals were primarily large hospitals selected based on their capacity to enroll a substantial number of patients and maintain high-quality surveillance. All patients with injuries presenting to the emergency departments of the participating hospitals during the surveillance period were included, rather than being sampled within hospitals. Data were collected according to standardized surveillance guidelines by trained coordinators using prehospital records, patient interviews, and medical records, and patients were followed from ED presentation through ED disposition or hospital discharge. Because the participating hospitals were not selected through probability sampling, the surveillance data may not be generalizable to the entire population of patients with injuries presenting to EDs in Korea.

**Figure 1:**
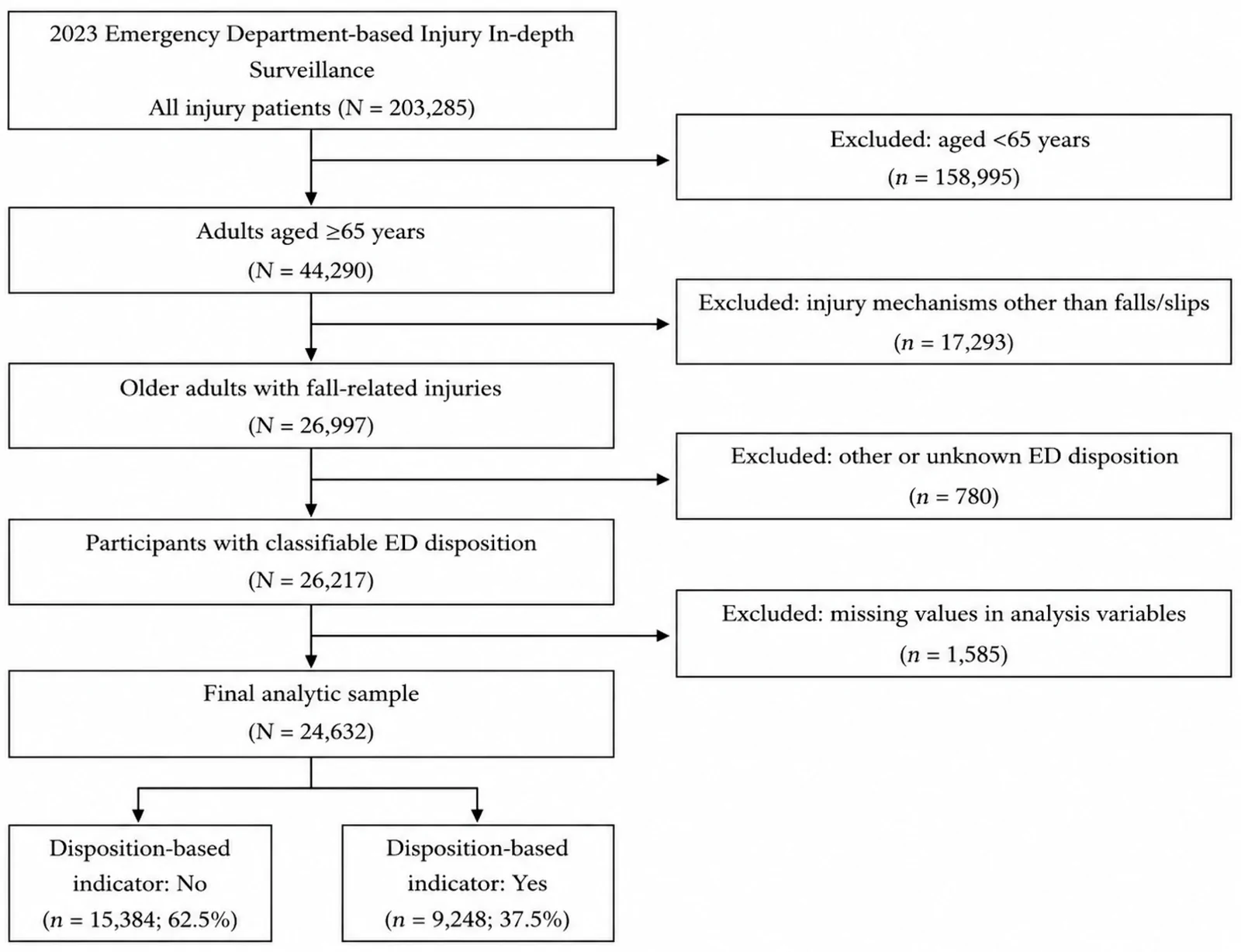
Flow diagram of participant selection and study outcome classification.

Of the 203,285 patients with injuries registered in the surveillance database, 44,290 were aged ≥65 years. Among them, 26,997 patients whose injury mechanism was classified as a fall or slip (fall-related injury) were selected. Subsequently, 780 patients with ED dispositions that could not be consistently classified according to the primary outcome definition were excluded: discharge because of terminal illness (*n* = 7), discharge without further curative treatment because recovery was considered unlikely (*n* = 2), transfer to a long-term care facility (*n* = 20), transfer at the request of the patient or guardian (*n* = 623), transfer for other reasons (*n* = 94), other dispositions (*n* = 33), and unknown disposition (*n* = 1). An additional 1585 patients (6.0%) with missing values in the analysis variables were excluded. Missingness was primarily observed for alcohol use at the time of injury (*n* = 1476, 5.6%), whereas missingness for the other analysis variables was minimal (≤0.2%). The final analytic sample consisted of 24,632 participants (Figure 1).

### 2.2. Variables

The dependent variable was a disposition-based indicator of severe injury. Based on ED disposition, patients who were discharged home or transferred to primary or secondary healthcare facilities for minor injuries were classified as the non-severe group. Patients who were admitted, transferred because of a lack of general or intensive care beds or inability to provide emergency surgery, transferred to a higher-level hospital for specialized emergency care, or died were classified as meeting the criteria for the disposition-based indicator of severe injury. Patients discharged because of terminal illness, discharged without further curative treatment because recovery was considered unlikely, transferred to long-term care facilities, transferred at the patient’s request, transferred for other reasons, or with unknown disposition were excluded from the analysis. ED disposition and hospital admission have been used as clinically relevant outcomes in injury research, and injury severity has been reported to be associated with hospital admission among older adults with fall-related injuries [16,17]. Therefore, the outcome was defined as a disposition-based indicator rather than a direct anatomical or physiological measure of injury severity.

Independent variables were selected based on factors considered in previous studies of fall-related injuries among older adults in relation to injury severity or ED disposition, as well as variables available in the Emergency Department-based Injury In-depth Surveillance, taking into account their clinical and epidemiological relevance [13,14]. The analysis included sex, age group, alcohol use at the time of injury, type of fall, injury location, indoor/outdoor setting, activity at the time of injury, time of injury, mode of transportation, and level of consciousness. Age was categorized into three groups: 65–74, 75–84, and ≥85 years. Alcohol use at the time of injury was defined using the alcohol-related status variable in the surveillance data. “No evidence of alcohol use” was classified as no alcohol use, whereas “patient alcohol use” or “both patient and related-person alcohol use” was classified as alcohol use; cases with no available information or alcohol use reported only for a related person were treated as missing. Type of fall was categorized as same-level falls, stair-related falls, or falls from a height. Injury location was recategorized from the original surveillance categories into home, healthcare or residential facilities, road or transportation areas, commercial or public facilities, and other locations; the latter included schools or educational facilities, sports facilities, industrial or construction facilities, farms or other primary-industry workplaces, outdoor areas such as the sea or rivers, and other specified locations. Indoor/outdoor setting was classified as indoors or outdoors. Activity at the time of injury was recategorized from the original surveillance categories into activities of daily living, sports or leisure activities, work-related activities, and other activities; the latter included education-related activities, medical treatment, travel, and other specified activities. Time of injury was classified as daytime or nighttime, and mode of transportation was categorized as non-emergency or emergency transportation. Level of consciousness was assessed using the AVPU (Alert, Voice, Pain, Unresponsive) scale. AVPU comprises four levels: A (Alert), V (responds to Voice), P (responds to Pain), and U (Unresponsive) [18]. In the primary analysis, A was classified as alert, whereas V, P, and U were combined and classified as impaired consciousness.

### 2.3. Statistical Analysis

Data were analyzed using IBM SPSS Statistics version 25.0 (IBM Corp., Armonk, NY, USA), with statistical significance set at *p* < 0.05. General characteristics, injury-related characteristics, and healthcare utilization and clinical characteristics were summarized using frequencies and percentages. Differences in participant characteristics according to the primary outcome were analyzed using the chi-square test. Multivariable logistic regression analysis was performed to identify factors associated with the primary outcome, and the results are presented as adjusted odds ratios (aORs) with 95% confidence intervals (CIs). Participants with missing data for one or more variables included in the primary multivariable analysis were excluded, and the primary analysis was conducted using complete cases. To assess potential differences associated with complete-case selection, available characteristics were compared between participants included in the complete-case analysis and those excluded because of missing values in one or more analysis variables using chi-square tests. Because this study was a secondary analysis of an established surveillance database, an a priori sample-size calculation was not performed. In the primary analysis, 9248 outcome events were available for 17 covariate coefficients, corresponding to approximately 544 events per covariate coefficient, indicating that the number of events was sufficient relative to the complexity of the multivariable model. Multicollinearity was assessed using tolerance and variance inflation factors (VIFs) after categorical variables included in the regression model were converted into dummy variables. The presence of influential individual observations was assessed using Cook’s influence statistics and leverage values. Model fit and explanatory power were evaluated using the Hosmer–Lemeshow goodness-of-fit test, Cox and Snell R^2^, and Nagelkerke R^2^, and overall classification accuracy was assessed using the classification table. Model discrimination was also evaluated using the receiver operating characteristic (ROC) curve, and the area under the curve (AUC) with its 95% CI was calculated.

Additional sensitivity analyses were conducted to assess the robustness of the findings. Two alternative disposition-based outcomes were defined as (1) hospital admission or transfer to a higher-level hospital for specialized emergency care and (2) death or transfer to a higher-level hospital for specialized emergency care, and multivariable logistic regression analyses were performed using the same independent variables as in the primary analysis. In addition, a sensitivity analysis using the Revised Trauma Score (RTS), a physiological measure of trauma severity, was conducted to assess injury severity from a perspective distinct from ED disposition, using a cutoff of ≤7.108 based on a previous mortality prediction study in older trauma patients [19]. Because the RTS includes the Glasgow Coma Scale as one of its components, level of consciousness was excluded as an independent variable from the RTS-based regression model. To assess whether dichotomizing AVPU into A versus V/P/U in the primary analysis might obscure differences across levels of consciousness impairment, an additional sensitivity analysis was conducted using the original four AVPU categories: A (Alert), V (responds to Voice), P (responds to Pain), and U (Unresponsive). In addition, patients discharged because of terminal illness or without further curative treatment because recovery was considered unlikely were classified as meeting the disposition-based severe injury indicator, and the multivariable logistic regression analysis was repeated. Because level of consciousness and mode of transportation may represent potential post-injury or downstream factors related to injury severity, an additional multivariable logistic regression analysis was performed after excluding these two variables from the model. Finally, given the relatively high prevalence of the primary outcome, a modified Poisson regression model with robust variance was fitted using the same covariates as in the primary analysis to estimate adjusted prevalence ratios (aPRs) and 95% CIs.

### 2.4. Ethical Considerations

This study was approved by the Institutional Review Board of Nambu University (No. 1041478-2026-HR-016; 12 June 2026). Following IRB approval, authorization to use the de-identified 2023 Emergency Department-based Injury In-depth Surveillance data was obtained from the Korea Disease Control and Prevention Agency, and the data were accessed on 22 June 2026. Because this study involved secondary analysis of de-identified data without direct contact with participants, informed consent was not applicable.

## 3. Results

### 3.1. Characteristics According to the Disposition-Based Severe Injury Indicator Among Older Adults with Fall-Related Injuries

Of the 26,217 participants with an eligible ED disposition, 1585 (6.0%) were excluded because of missing values in one or more analysis variables, leaving 24,632 participants in the complete-case analysis. Compared with participants included in the complete-case analysis, those excluded because of missing data differed significantly in age group, type of fall, activity at the time of injury, mode of transportation, and time of injury (all *p* < 0.05), whereas no significant differences were observed in sex, location of injury, indoor/outdoor setting, level of consciousness, or the disposition-based severe injury indicator. The proportion meeting the criteria for the disposition-based severe injury indicator was similar between the excluded and included groups (38.4% vs. 37.5%, respectively; *p* = 0.484).

Among the 24,632 participants included in the final analysis, 15,384 (62.5%) did not meet the criteria for the disposition-based indicator of severe injury, whereas 9248 (37.5%) met the criteria. Among the latter, 8806 (35.7% of the total sample) were admitted; 322 (1.3%) were transferred because of limited hospital capacity, inability to provide immediate emergency surgery or treatment, or the need for specialized emergency care at a higher-level hospital; and 120 (0.5%) died in the ED. Comparisons of participant characteristics according to the disposition-based severe injury indicator showed significant differences in age group, alcohol use at the time of injury, type of fall, location of injury, indoor/outdoor setting, activity at the time of injury, mode of transportation, time of injury, and level of consciousness, but not in sex (Table 1).

The proportion meeting the criteria for the disposition-based severe injury indicator increased with age, from 32.9% among those aged 65–74 years to 38.9% among those aged 75–84 years and 42.6% among those aged ≥85 years (χ^2^ = 150.45, *p* < 0.001). The proportion was higher among participants who had not consumed alcohol at the time of injury than among those who had (38.7% vs. 20.1%; χ^2^ = 220.59, *p* < 0.001). By type of fall, the proportion was highest for falls from a height (50.5%), followed by same-level falls (36.2%) and stair-related falls (31.3%; χ^2^ = 280.06, *p* < 0.001). By location of injury, the proportions were 45.8% for other locations, 43.1% for medical or residential facilities, 41.4% for home, 29.2% for commercial or public facilities, and 27.7% for road or traffic areas (χ^2^ = 461.24, *p* < 0.001). The proportion was higher for injuries occurring indoors than for those occurring outdoors (39.9% vs. 34.1%; χ^2^ = 83.67, *p* < 0.001).

By activity at the time of injury, the proportion was highest for work-related activities (44.4%), followed by activities of daily living (39.0%), other activities (33.8%), and sports or leisure activities (28.9%; χ^2^ = 209.69, *p* < 0.001). The proportion was higher for daytime injuries than for nighttime injuries (38.7% vs. 35.3%; χ^2^ = 26.85, *p* < 0.001). Participants transported by emergency transportation had a higher proportion than those transported by non-emergency transportation (48.2% vs. 23.6%; χ^2^ = 1560.60, *p* < 0.001), and participants with impaired consciousness had a higher proportion than those who were alert (69.9% vs. 35.6%; χ^2^ = 644.60, *p* < 0.001).

### 3.2. Factors Associated with the Disposition-Based Severe Injury Indicator Among Older Adults with Fall-Related Injuries

Multivariable logistic regression analysis was performed to identify factors associated with the disposition-based indicator of severe injury among older adults with fall-related injuries (Table 2). The overall regression model was statistically significant (χ^2^ = 2854.106, df = 17, *p* < 0.001). The Cox and Snell R^2^ was 0.109, the Nagelkerke R^2^ was 0.149, and the overall classification accuracy was 66.8%. The Hosmer–Lemeshow goodness-of-fit test was statistically significant (χ^2^ = 54.459, df = 8, *p* < 0.001). Multicollinearity diagnostics showed tolerance values ranging from 0.706 to 0.955 and VIF values ranging from 1.047 to 1.416, indicating no problematic multicollinearity among the independent variables. Cook’s influence statistics ranged from 0.00002 to 0.00606, and leverage values ranged from 0.00018 to 0.00258, with no evidence of highly influential individual observations. ROC analysis yielded an AUC of 0.695 (95% CI = 0.688–0.701), indicating limited discriminative ability of the model.

Compared with participants aged 65–74 years, those aged 75–84 years (aOR = 1.141, 95% CI = 1.069–1.217, *p* < 0.001) and those aged ≥85 years (aOR = 1.133, 95% CI = 1.048–1.224, *p* = 0.002) had higher odds of meeting the disposition-based severe injury indicator. Participants who had consumed alcohol at the time of injury had lower odds than those who had not (aOR = 0.410, 95% CI = 0.355–0.473, *p* < 0.001). Regarding type of fall, compared with same-level falls, falls from a height were associated with higher odds (aOR = 1.318, 95% CI = 1.210–1.435, *p* < 0.001), whereas stair-related falls were associated with lower odds (aOR = 0.792, 95% CI = 0.717–0.875, *p* < 0.001). Regarding location of injury, compared with home, injuries occurring in road or traffic areas (aOR = 0.612, 95% CI = 0.554–0.675, *p* < 0.001) and commercial or public facilities (aOR = 0.706, 95% CI = 0.630–0.792, *p* < 0.001) were associated with lower odds, whereas injuries occurring in other locations were associated with higher odds (aOR = 1.159, 95% CI = 1.018–1.321, *p* = 0.026). Injuries occurring outdoors were associated with higher odds than those occurring indoors (aOR = 1.116, 95% CI = 1.027–1.213, *p* = 0.009).

Regarding activity at the time of injury, compared with activities of daily living, sports or leisure activities (aOR = 0.838, 95% CI = 0.767–0.916, *p* < 0.001) and other activities (aOR = 0.803, 95% CI = 0.692–0.932, *p* = 0.004) were associated with lower odds. Nighttime injuries were associated with lower odds than daytime injuries (aOR = 0.865, 95% CI = 0.814–0.918, *p* < 0.001). Participants transported by emergency transportation had higher odds than those transported by non-emergency transportation (aOR = 2.910, 95% CI = 2.746–3.085, *p* < 0.001), and participants with impaired consciousness had higher odds than those who were alert (aOR = 3.340, 95% CI = 2.942–3.791, *p* < 0.001). Sex was not significantly associated with the disposition-based severe injury indicator after adjustment (aOR = 0.958, 95% CI = 0.904–1.016, *p* = 0.151).

### 3.3. Sensitivity Analyses of Factors Associated with the Disposition-Based Severe Injury Indicator Among Older Adults with Fall-Related Injuries

To assess the robustness of the main findings, additional sensitivity analyses were conducted using alternative outcome definitions, a physiological measure of trauma severity, alternative classification of level of consciousness, different covariate specifications, and an alternative regression model. When hospital admission or transfer to a higher-level hospital for specialized emergency care was defined as the outcome, 8843 participants (35.9%) met the criteria (Table 3). Emergency transportation (aOR = 2.979, 95% CI = 2.809–3.160), impaired consciousness (aOR = 2.287, 95% CI = 2.030–2.577), and falls from a height (aOR = 1.214, 95% CI = 1.115–1.323) showed higher odds, whereas alcohol use at the time of injury showed lower odds (aOR = 0.440, 95% CI = 0.381–0.508).

When death or transfer to a higher-level hospital for specialized emergency care was defined as the outcome, 157 participants (0.6%) met the criteria. Impaired consciousness (aOR = 16.259, 95% CI = 11.066–23.889), emergency transportation (aOR = 4.330, 95% CI = 2.136–8.780), falls from a height (aOR = 3.885, 95% CI = 2.535–5.955), and injuries occurring outdoors (aOR = 3.216, 95% CI = 2.009–5.146) showed higher odds.

Among the 24,477 participants with available RTS data, 845 (3.5%) had an RTS ≤ 7.108. In the analysis excluding level of consciousness from the independent variables, emergency transportation (aOR = 7.021, 95% CI = 5.571–8.848), falls from a height (aOR = 2.129, 95% CI = 1.767–2.566), stair-related falls (aOR = 1.645, 95% CI = 1.322–2.048), and injuries occurring outdoors (aOR = 1.341, 95% CI = 1.105–1.628) showed higher odds.

When the original four AVPU categories were analyzed, with A (Alert) as the reference category, the aOR was 1.812 (95% CI = 1.565–2.098) for V (responds to Voice), 14.509 (95% CI = 9.764–21.560) for P (responds to Pain), and 71.114 (95% CI = 26.252–192.642) for U (Unresponsive). When patients discharged because of terminal illness (*n* = 7) or without further curative treatment because recovery was considered unlikely (*n* = 2) were additionally classified as meeting the disposition-based severe injury indicator, the results of the multivariable analysis remained essentially unchanged from those of the primary analysis.

In addition, when level of consciousness and mode of transportation were excluded as potential post-injury or downstream factors, the direction of associations for the remaining covariates was generally consistent with that observed in the primary analysis. Finally, the modified Poisson regression analysis with robust variance showed an overall pattern of associations generally consistent with that of the primary logistic regression analysis, although the magnitudes of several associations were attenuated. Emergency transportation (aPR = 1.962, 95% CI = 1.887–2.040), impaired consciousness (aPR = 1.608, 95% CI = 1.545–1.674), and falls from a height (aPR = 1.135, 95% CI = 1.089–1.183) remained positively associated with the disposition-based severe injury indicator.

## 4. Discussion

This study examined factors associated with the disposition-based indicator of severe injury among older adults with fall-related injuries using data from the 2023 Emergency Department-based Injury In-depth Surveillance. Multivariable logistic regression analysis showed that age group, alcohol use at the time of injury, type of fall, location of injury, indoor/outdoor setting, activity at the time of injury, mode of transportation to the ED, time of injury, and level of consciousness were significantly associated with the disposition-based indicator of severe injury. However, given the large sample size, even associations of modest magnitude may reach statistical significance. Therefore, individual findings should be interpreted cautiously by considering the magnitude of the associations and their 95% CIs, rather than statistical significance alone.

In this study, compared with adults aged 65–74 years, those aged 75–84 years and ≥85 years had higher odds of meeting the disposition-based indicator of severe injury, although the magnitude of these associations was modest. In a study of older trauma patients in Korea, age, along with injury severity and the need for early transfusion, was incorporated into a geriatric trauma prognostic score for trauma-related mortality, and the score was reported to be useful for predicting in-hospital mortality [20]. In another study of older trauma patients presenting to the emergency department, the hospitalization rate was higher among patients aged ≥80 years than among those aged 65–79 years, and femoral and pelvic fractures were also more common in patients aged ≥80 years [21]. Age-related declines in sensory and motor function and physiological reserve are known to increase vulnerability to trauma in older adults [22], and these characteristics may have contributed to the findings observed in the older age groups in the present study. However, sensitivity analyses showed that the association with age was not consistent across different outcome definitions. Therefore, the association between age and the disposition-based severe injury indicator observed in the primary analysis should not be interpreted as reflecting the effect of age alone; rather, age should be considered alongside other clinical characteristics when assessing older adults with fall-related injuries.

In this study, older adults who had consumed alcohol at the time of injury had lower odds of the disposition-based severe injury indicator than those who had not consumed alcohol. Previous studies have reported that recurrent binge drinking is associated with an increased risk of fall-related injuries among older adults and that head injuries and traumatic brain injuries are more common among patients with alcohol-related falls [23,24]. A previous study using the Korean Emergency Department-based Injury In-depth Surveillance data also reported that, among patients with alcohol-related falls, male sex, daytime injury, ambulance transportation, injury in residential facilities, and slippery surfaces were associated with severe injury [14]. However, direct comparison with these studies is limited because of differences in outcome definitions and analytical approaches. The alcohol variable in the present study reflected only alcohol use at the time of injury and did not capture the amount or usual pattern of alcohol consumption; moreover, restricting the analysis to patients included in an ED-based surveillance system may have introduced measurement and selection biases. Alcohol use was also not significantly associated with physiological severity in the RTS-based analysis. Therefore, the observed inverse association should not be interpreted as a protective effect of alcohol and should be interpreted cautiously in light of potential selection bias, measurement limitations, and residual confounding by unmeasured factors. Future studies should examine the association between alcohol use and outcomes of fall-related injuries among older adults while considering the amount and pattern of alcohol consumption, fall mechanisms, and clinical characteristics.

In this study, compared with same-level falls, falls from a height were associated with higher odds of meeting the disposition-based indicator of severe injury, whereas stair-related falls were associated with lower odds. Previous studies have reported that falls in older adults can result in serious injuries, including head injuries and hip fractures, and may lead to hospitalization and death [12]. A study using emergency medical services data also reported that patterns of injury and trauma severity may vary according to the environment in which falls occur [11]. Taken together, these findings suggest that fall mechanisms and environmental circumstances may be associated with injury outcomes among older adults. However, although stair-related falls were associated with lower odds of the disposition-based severe injury indicator than same-level falls in the primary analysis, they were associated with higher odds of physiological injury severity in the sensitivity analysis using RTS ≤7.108. Therefore, the lower odds observed for stair-related falls should not be interpreted as indicating lower injury severity. Clinical disposition, such as hospital admission or transfer, may be influenced not only by injury severity but also by the patient’s clinical condition and circumstances of care. In addition, detailed information on the circumstances of the fall, such as fall height and structural characteristics of stairs, was not available in this study. Future studies should further differentiate fall mechanisms and incorporate anatomical or physiological measures of injury severity to clarify the association between fall type and injury outcomes.

In this study, compared with falls occurring at home, falls occurring in road or transportation areas and commercial or public facilities were associated with lower odds of meeting the disposition-based indicator of severe injury, whereas falls occurring in other locations were associated with higher odds. Previous research has also reported that outcomes among older adults with fall-related injuries may vary according to the location of injury [9]. However, because the outcome in the present study was a disposition-based indicator comprising hospital admission, transfer to a higher-level hospital, and death, these differences across injury locations should not be interpreted as directly reflecting differences in injury severity itself.

In this study, falls occurring outdoors were associated with modestly higher odds of meeting the disposition-based indicator of severe injury than falls occurring indoors. A study using emergency medical services data similarly reported that the distribution of trauma severity differed between indoor and outdoor falls according to age and sex [11]. Various environmental factors, including roads, stairs, and slopes, may influence injury patterns in outdoor settings; however, detailed information on these environmental characteristics was not available in the present study. Therefore, the association between outdoor falls and the disposition-based severe injury indicator should not be interpreted as an effect of the outdoor setting itself but rather should be interpreted cautiously in the context of the specific fall environment and injury mechanism.

In this study, compared with injuries occurring during activities of daily living, injuries occurring during sports or leisure activities and other activities were associated with lower odds of meeting the disposition-based indicator of severe injury. Previous research has described the characteristics of older adults with fall-related injuries by categorizing activities at the time of the fall into activities of daily living, mobility, work-related activities, and activities related to treatment; however, the independent association between activity type and mortality or injury severity has not been sufficiently established [9]. Therefore, the differences observed across activity types in the present study should not be interpreted as effects of the activities themselves, as the environment in which the activity occurred, the fall mechanism, and the health and functional characteristics of the individual may also have contributed to these findings.

In this study, injuries occurring at night were associated with modestly lower odds of meeting the disposition-based indicator of severe injury than those occurring during the daytime. A previous study of older adults with alcohol-related falls also reported that daytime injuries were associated with severe injury [14]; however, the study population differed from that of the present study because it was limited to patients with alcohol-related falls. In the sensitivity analyses, time of injury was not significantly associated with death or transfer to a higher-level hospital or with RTS-based physiological injury severity. Therefore, the lower odds observed for nighttime injuries in the primary analysis should not be interpreted as an effect of time of injury itself and should be interpreted cautiously, considering characteristics that may vary by time of injury, such as activity type, location of the fall, alcohol use, and patterns of healthcare utilization.

In this study, patients transported by emergency medical services had higher odds of the disposition-based severe injury indicator than those using non-emergency transportation. Previous studies have reported that emergency medical services play an important role in the prehospital assessment and transportation of older adults with fall-related injuries [25], and ambulance transportation has been associated with hospital admission among older patients with falls [26]. In addition, clinical information collected in the prehospital setting may be useful for assessing trauma severity in injured patients [11]. In the present study, emergency transportation was consistently associated with higher odds of the primary outcome in the primary analysis as well as in sensitivity analyses using alternative disposition-based outcomes and the RTS. However, patients with greater injury severity may be more likely both to receive emergency transportation and to be admitted or transferred, potentially resulting in confounding by indication; collider bias also cannot be excluded because the analysis was restricted to patients presenting to participating EDs. Therefore, emergency transportation should be interpreted as a marker of perceived injury severity and initial clinical status rather than as a cause of adverse disposition.

In this study, patients with impaired consciousness had higher odds of meeting the disposition-based indicator of severe injury than those who were alert. Previous studies have reported that AVPU is highly correlated with GCS and can be used for the rapid assessment of level of consciousness in prehospital and emergency department settings [27,28]. A recent systematic review and meta-analysis of older trauma patients also identified GCS as one of the major injury-related factors associated with mortality [29]. In the present study, impaired consciousness was also associated with higher odds in sensitivity analyses using alternative disposition-based outcomes. Furthermore, when the original four AVPU categories were analyzed, the magnitude of the association with the disposition-based indicator of severe injury increased as the level of consciousness deteriorated, with A (Alert) as the reference category. These findings indicate that combining V, P, and U into a single impaired-consciousness category may not fully capture differences according to the degree of consciousness impairment. However, because impaired consciousness reflects the clinical status of an injury that has already occurred, it should not be interpreted as a cause of adverse clinical disposition but rather as information reflecting the initial clinical status of older adults with fall-related injuries. These findings do not demonstrate that AVPU provides incremental predictive value beyond established measures such as the GCS or trauma severity scores, and its potential clinical utility in this context requires further investigation.

This study has several limitations inherent to the secondary analysis of data from the Emergency Department-based Injury In-depth Surveillance. First, the primary outcome was a disposition-based indicator rather than a direct measure of anatomical injury severity. Admission or transfer may be influenced not only by injury severity but also by patients’ clinical status and institutional practices and transfer capacity. In addition, some exposures and outcomes defined or recategorized using surveillance data may have been subject to measurement error or misclassification. Although sensitivity analyses using alternative disposition-based outcomes and the RTS were conducted to address this limitation, the direction and magnitude of some associations varied across outcome definitions. Second, information on comorbidities, frailty, functional status, and medication use was unavailable; therefore, these potentially relevant clinical factors could not be included in the analyses, and residual confounding by unmeasured factors cannot be excluded. Third, the primary analysis was restricted to complete cases, excluding 1585 participants (6.0%) with missing data on analysis variables, which may have introduced selection bias. In addition, 155 participants without RTS data were excluded from the RTS-based analysis, and the analysis of death or transfer to a higher-level hospital included only 157 events, warranting caution regarding the precision of these estimates. Fourth, the surveillance data were derived from 23 participating hospitals rather than a nationally representative probability sample; therefore, the findings may not be generalizable to all older adults with fall-related injuries presenting to EDs in Korea. Hospital-level clustering was not explicitly accounted for, and residual heterogeneity across hospitals may remain. Furthermore, the findings were not externally validated and should therefore be applied cautiously to other populations or healthcare settings. Finally, because this study was a retrospective secondary analysis of observational data, temporal relationships between variables could not be clearly established, and the observed associations should not be interpreted as causal. Despite these limitations, this study analyzed characteristics associated with ED disposition among older adults with fall-related injuries using multicenter ED surveillance data and assessed the robustness of the main findings through sensitivity analyses using alternative disposition-based outcomes and a physiological measure of injury severity. Future prospective studies incorporating anatomical injury severity, comorbidities, frailty, functional status, and medication use, as well as external validation, are warranted.

## 5. Conclusions

This study analyzed factors associated with a disposition-based severe injury indicator among older adults with fall-related injuries using data from the 2023 Emergency Department-based Injury In-depth Surveillance. Several sociodemographic, injury-related, and initial clinical characteristics were statistically associated with the disposition-based severe injury indicator. In particular, emergency transportation and impaired consciousness showed strong associations; however, these findings should not be interpreted as causal effects but rather as characteristics reflecting perceived injury severity and initial clinical status at the time of injury. Sensitivity analyses also showed that the magnitude and direction of some associations varied depending on the outcome definition and analytical approach. These findings suggest that patient and injury characteristics, together with initial clinical status, should be considered comprehensively when interpreting ED outcomes among older adults with fall-related injuries.

## Figures and Tables

**Table 1:** Characteristics According to the Disposition-Based Severe Injury Indicator Among Older Adults with Fall-Related Injuries (*N* = 24,632).

Variable	Category	Total *n* (%)	Indicator: No *n* (%)	Indicator: Yes *n* (%)	χ^2^	*p*
Sex	Female	14,369 (58.3)	8920 (62.1)	5449 (37.9)	2.09	0.148
	Male	10,263 (41.7)	6464 (63.0)	3799 (37.0)		
Age group (yr.)	65–74	9056 (36.8)	6073 (67.1)	2983 (32.9)	150.45	<0.001
	75–84	10,094 (41.0)	6166 (61.1)	3928 (38.9)		
	≥85	5482 (22.3)	3145 (57.4)	2337 (42.6)		
Alcohol use at the time of injury	No	23,044 (93.6)	14,115 (61.3)	8929 (38.7)	220.59	<0.001
	Yes	1588 (6.4)	1269 (79.9)	319 (20.1)		
Type of fall	Same-level fall	19,038 (77.3)	12,146 (63.8)	6892 (36.2)	280.06	<0.001
	Stair-related fall	2445 (9.9)	1679 (68.7)	766 (31.3)		
	Fall from a height	3149 (12.8)	1559 (49.5)	1590 (50.5)		
Location of injury	Home	13,549 (55.0)	7942 (58.6)	5607 (41.4)	461.24	<0.001
	Medical or residential facilities	1363 (5.5)	775 (56.9)	588 (43.1)		
	Road or traffic areas	5844 (23.7)	4227 (72.3)	1617 (27.7)		
	Commercial or public facilities	2046 (8.3)	1449 (70.8)	597 (29.2)		
	Other	1830 (7.4)	991 (54.2)	839 (45.8)		
Indoor or outdoor setting	Indoor	14,689 (59.6)	8833 (60.1)	5856 (39.9)	83.67	<0.001
	Outdoor	9943 (40.4)	6551 (65.9)	3392 (34.1)		
Activity at the time of injury	Activities of daily living	16,670 (67.7)	10,172 (61.0)	6498 (39.0)	209.69	<0.001
	Sports or leisure activities	4318 (17.5)	3070 (71.1)	1248 (28.9)		
	Work-related activities	2555 (10.4)	1421 (55.6)	1134 (44.4)		
	Other activities	1089 (4.4)	721 (66.2)	368 (33.8)		
Time of injury	Daytime	16,116 (65.4)	9878 (61.3)	6238 (38.7)	26.85	<0.001
	Nighttime	8516 (34.6)	5506 (64.7)	3010 (35.3)		
Mode of transportation to the emergency department	Non-emergency transportation	10,644 (43.2)	8135 (76.4)	2509 (23.6)	1560.60	<0.001
	Emergency transportation	13,988 (56.8)	7249 (51.8)	6739 (48.2)		
Level of consciousness (AVPU)	Alert	23,265 (94.5)	14,972 (64.4)	8293 (35.6)	644.60	<0.001
	Impaired consciousness *	1367 (5.5)	412 (30.1)	955 (69.9)		

* Note. Values are presented as *n* (%). Indicator: No and Indicator: Yes indicate whether participants did not meet or met the criteria for the disposition-based severe injury indicator, respectively. Percentages in these columns represent row percentages within each category. *p*-values were obtained using the chi-square test. ED, emergency department. Impaired consciousness was defined as responsiveness to voice, responsiveness to pain, or unresponsiveness (V, P, or U on the AVPU scale).

**Table 2:** Factors Associated with the Disposition-Based Severe Injury Indicator Among Older Adults with Fall-Related Injuries.

Variable	Adjusted OR	95% CI	*p*
**Sex**			
Male (vs. Female)	0.958	0.904–1.016	0.151
**Age group (yr.)**			
75–84 (vs. 65–74)	1.141	1.069–1.217	<0.001
≥85 (vs. 65–74)	1.133	1.048–1.224	0.002
**Alcohol use at the time of injury**			
Yes (vs. No)	0.410	0.355–0.473	<0.001
**Type of fall**			
Stair-related fall (vs. Same-level fall)	0.792	0.717–0.875	<0.001
Fall from a height (vs. Same-level fall)	1.318	1.210–1.435	<0.001
**Location of injury**			
Medical or residential facilities (vs. Home)	1.014	0.894–1.149	0.830
Road or traffic areas (vs. Home)	0.612	0.554–0.675	<0.001
Commercial or public facilities (vs. Home)	0.706	0.630–0.792	<0.001
Other (vs. Home)	1.159	1.018–1.321	0.026
**Indoor or outdoor setting**			
Outdoor (vs. Indoor)	1.116	1.027–1.213	0.009
**Activity at the time of injury**			
Sports or leisure activities (vs. Activities of daily living)	0.838	0.767–0.916	<0.001
Work-related activities (vs. Activities of daily living)	1.071	0.965–1.188	0.196
Other activities (vs. Activities of daily living)	0.803	0.692–0.932	0.004
**Time of injury**			
Nighttime (vs. Daytime)	0.865	0.814–0.918	<0.001
**Mode of transportation to the emergency department**			
Emergency transportation (vs. Non-emergency transportation)	2.910	2.746–3.085	<0.001
**Level of consciousness (AVPU)**			
Impaired consciousness (vs. Alert)	3.340	2.942–3.791	<0.001

Note. aOR, adjusted odds ratio; CI, confidence interval; ED, emergency department. Adjusted odds ratios were estimated using a multivariable logistic regression model including sex, age group, alcohol use at the time of injury, type of fall, location of injury, indoor/outdoor setting, activity at the time of injury, time of injury, mode of transportation to the emergency department, and level of consciousness. Nagelkerke R^2^ = 0.149; Cox and Snell R^2^ = 0.109; −2 log likelihood = 29,748.362.

**Table 3:** Sensitivity Analyses of Factors Associated with the Disposition-Based Severe Injury Indicator Among Older Adults with Fall-Related Injuries.

Variables	Admission or Higher-Level Transfer aOR (95% CI)	Death or Higher-Level Transfer aOR (95% CI)	RTS ≤ 7.108 aOR (95% CI)	Primary Outcome with Four-Category AVPU aOR (95% CI)
**Sex**				
Female	Ref.	Ref.	Ref.	Ref.
Male	0.968 (0.913–1.026)	1.531 (1.035–2.265)	1.956 (1.682–2.274)	0.946 (0.893–1.003)
**Age group (yr.)**				
65–74	Ref.	Ref.	Ref.	Ref.
75–84	1.152 (1.080–1.230)	1.069 (0.697–1.641)	0.886 (0.752–1.043)	1.150 (1.077–1.227)
≥85	1.159 (1.072–1.252)	1.334 (0.806–2.209)	0.738 (0.600–0.907)	1.149 (1.063–1.242)
**Alcohol use at the time of injury**				
No	Ref.	Ref.	Ref.	Ref.
Yes	0.440 (0.381–0.508)	0.545 (0.215–1.380)	1.142 (0.872–1.494)	0.410 (0.354–0.474)
**Type of fall**				
Same-level fall	Ref.	Ref.	Ref.	Ref.
Stair-related fall	0.817 (0.740–0.903)	0.819 (0.387–1.735)	1.645 (1.322–2.048)	0.777 (0.702–0.859)
Fall from a height	1.214 (1.115–1.323)	3.885 (2.535–5.955)	2.129 (1.767–2.566)	1.295 (1.188–1.411)
**Location of injury**				
Home	Ref.	Ref.	Ref.	Ref.
Medical/residential facilities	1.087 (0.959–1.232)	0.837 (0.448–1.566)	1.450 (1.096–1.919)	1.030 (0.909–1.169)
Road/traffic areas	0.657 (0.595–0.725)	0.293 (0.159–0.539)	0.585 (0.458–0.747)	0.626 (0.567–0.692)
Commercial/public facilities	0.704 (0.627–0.790)	0.426 (0.171–1.058)	0.719 (0.539–0.958)	0.707 (0.630–0.794)
Other	1.222 (1.073–1.392)	0.511 (0.258–1.010)	0.841 (0.619–1.143)	1.187 (1.041–1.353)
**Indoor/outdoor setting**				
Indoor	Ref.	Ref.	Ref.	Ref.
Outdoor	1.037 (0.954–1.127)	3.216 (2.009–5.146)	1.341 (1.105–1.628)	1.100 (1.012–1.197)
**Activity at the time of injury**				
Activities of daily living	Ref.	Ref.	Ref.	Ref.
Sports/leisure activities	0.833 (0.761–0.911)	0.752 (0.376–1.502)	0.918 (0.732–1.152)	0.820 (0.750–0.897)
Work-related activities	1.081 (0.974–1.199)	0.695 (0.364–1.329)	0.824 (0.631–1.074)	1.060 (0.956–1.177)
Other activities	0.618 (0.530–0.721)	5.293 (3.154–8.882)	1.370 (0.991–1.894)	0.771 (0.661–0.898)
**Time of injury**				
Daytime	Ref.	Ref.	Ref.	Ref.
Nighttime	0.857 (0.807–0.911)	0.758 (0.503–1.141)	1.020 (0.875–1.190)	0.864 (0.814–0.918)
**Mode of transportation to the emergency department**				
Non-emergency transportation	Ref.	Ref.	Ref.	Ref.
Emergency transportation	2.979 (2.809–3.160)	4.330 (2.136–8.780)	7.021 (5.571–8.848)	2.884 (2.721–3.056)
**Level of consciousness (AVPU)**				
A (Alert)	Ref.	Ref.	— ^a^	Ref.
Impaired consciousness (V/P/U)	2.287 (2.030–2.577)	16.259 (11.066–23.889)	— ^a^	—
V (responds to Voice)	—	—	—	1.812 (1.565–2.098)
P (responds to Pain)	—	—	—	14.509 (9.764–21.560)
U (Unresponsive)	—	—	—	71.114 (26.252–192.642)

Note. aOR, adjusted odds ratio; CI, confidence interval; RTS, Revised Trauma Score; AVPU, Alert, Voice, Pain, Unresponsive; Ref., reference category; —, not applicable. ^a^ Level of consciousness was excluded from the RTS-based model because neurological status is incorporated into the RTS.

## Data Availability

The data used in this study were obtained from the Korea Disease Control and Prevention Agency (KDCA) upon application and approval. The data are not publicly available and are subject to the KDCA’s data access and use procedures.
